# Special forms in twin pregnancy – asymmetric conjoined twins

**Published:** 2015

**Authors:** FA Anca, A Negru, AE Mihart, C Grigoriu, RE Bohîlțea

**Affiliations:** * ”Carol Davila” University of Medicine and Pharmacy, Bucharest, Romania; ** Department of Obstetrics and Gynecology, University Emergency Hospital Bucharest, Romania

**Keywords:** epigastric heteropagus, twin monozygous pregnancy, conjoined twins, asymmetrical twins, omphalocele

## Abstract

Twin pregnancies generally represent a high-risk pregnancy. However, monozygous twins are real challenges for obstetricians due to the complications that may occur. Among the particular cases of monozygous twins in the University Emergency Hospital of Bucharest, Department of Obstetrics and Gynecology, a monochorial monoamniotic pregnancy with conjoined twins has been described. These particular medical circumstances require a deeper understanding of the vascular anatomical particularities. An accurate diagnosis implies a most detailed description of the morphological dynamics of the fetuses with the study of the impact of the vascular anomaly on their development so that the maximum chances of survival and the best outcome for the viable fetus can be obtained. The diagnosis of the most frequently associated anomalies is also extremely important.

**Abbreviations:** MRI = Magnetic resonance imaging

## Case report

**Fig. 1:**
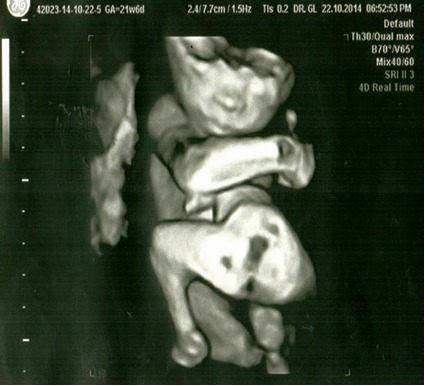
4D echography, gestational age 21 weeks, viable fetus and parasite fetus (face)

**Fig. 2:**
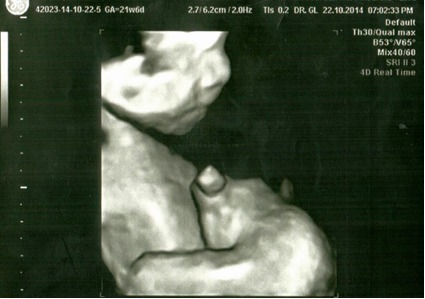
4D echography, gestational age 21 weeks, viable fetus and parasite fetus (profile)

**Fig. 3:**
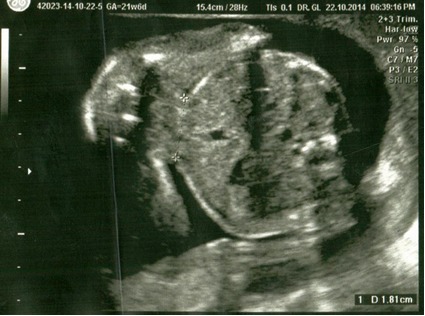
4D echography, gestational age 21 weeks, viable fetus and parasite fetus (measurement of anchor pedicle)

Patient aged 34, Caucasian, was admitted in the Department of Obstetrics and Gynecology of the University University Hospital of Bucharest for monitoring and ultrasonographic evaluation at 21 weeks of gestational age. Her personal obstetrical history revealed 5 vaginal live births without any complications, with normally developed fetuses. No twin pregnancies were recorded in the family history. An ultrasonographic diagnosis was established: monochorionic monoamniotic twin pregnancy, 21 weeks of gestational age, with one of the fetuses being an external parasite intimately attached to the epigastric region of the viable fetus, epigastric heteropagus, both male (**Fig. 1**-**3**).

**Fig. 4:**
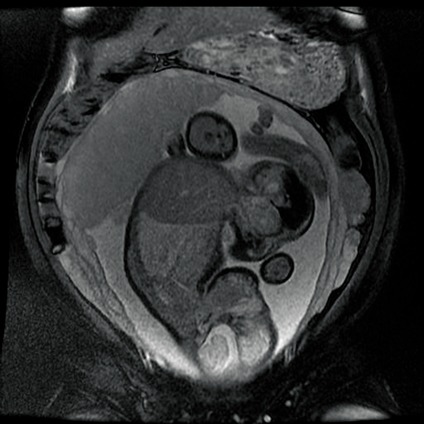
Fetal MRI–gestational age 21 weeks, viable fetus and parasite fetus (longitudinal plan)

**Fig. 5:**
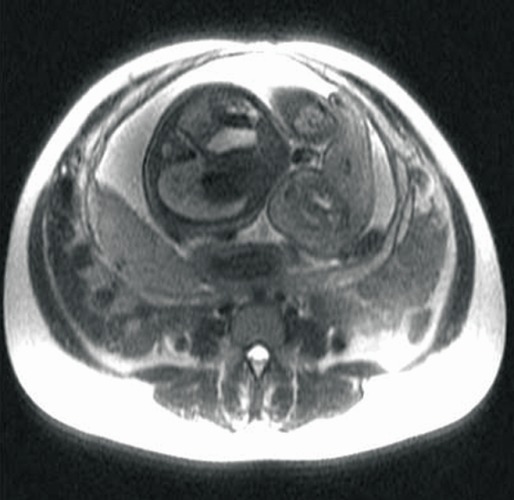
Fetal MRI–gestational age 22 weeks, viable fetus and parasite fetus (transverse plan)

**Fig. 6:**
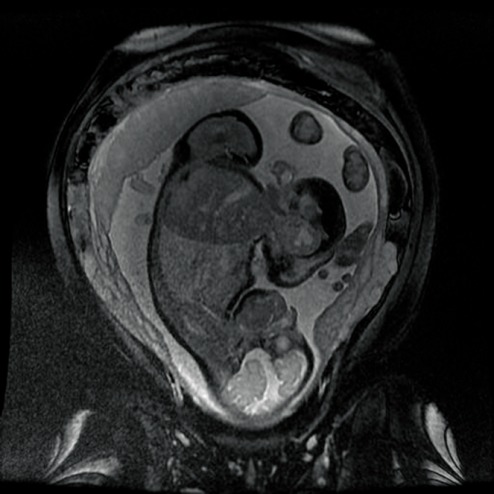
Fetal RMN–gestational age 22 weeks, viable fetus and parasite fetus

**Fig. 7:**
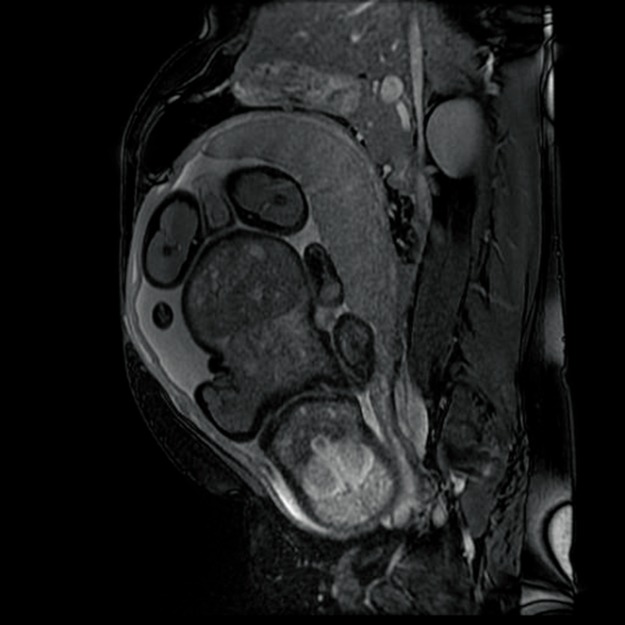
Fetal MRI–gestational age 22 weeks, viable fetus

In order to establish the vascular connection between the two fetuses, the patient had an MRI, which confirmed the ultrasonographic discoveries and revealed the parasitic fetus, partially developed, acephalic, with trunk and inferior members present and one kidney. The connection area between the two also displayed an omphalocele (**Fig. 4**-**7**). 

**Fig. 8:**
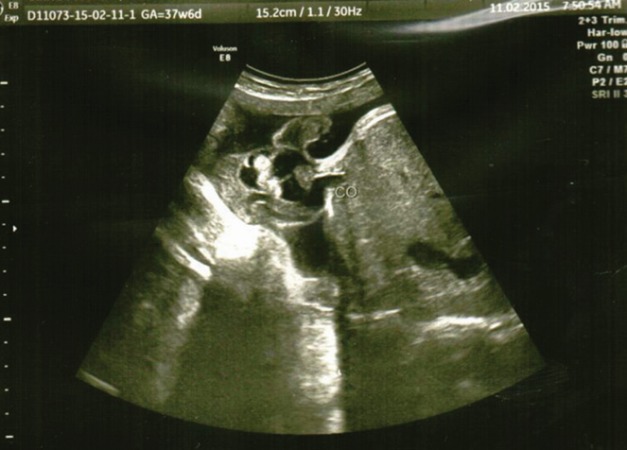
2D sonography–the insertion of the umbilical cord at the level of omphalocele to the viable fetus

The patient was further recommended medical and psychological support according to international protocols concerning the risks of the pregnancy and the necessity of amniocentesis, procedure that was refused by the patient. Further on, a multidisciplinary team was established, consisting of an obstetrician, a pediatric surgeon and a neonatologist in order to have a better monitoring of both mother and viable fetus. The patient was regularly examined ultrasonographically (**Fig. 8**) and laboratory tests were within normal range. 

At the gestational age of 38 weeks, the doctor decided that the patient delivered by segment-transverse cesarean prolonged in “T”. The delivered fetus was a male of 3600 grams, length of 51cm, with an external twin parasite, acephalic, upper limbs present, gluteal region and external genital organs upright, attached to the epigastrium of the well-developed fetus connected through a 50-millimeter implantation area (**Fig. 9a**,**Fig. 9b**). 

**Fig. 9a:**
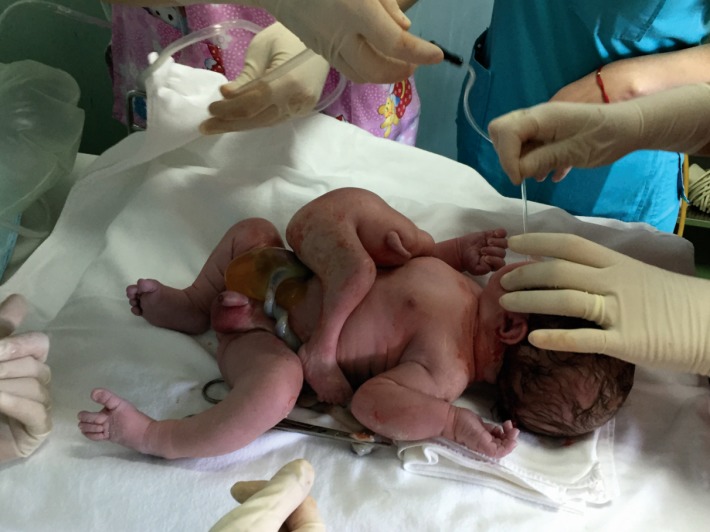
Asymmetric conjoined twins

**Fig. 9b:**
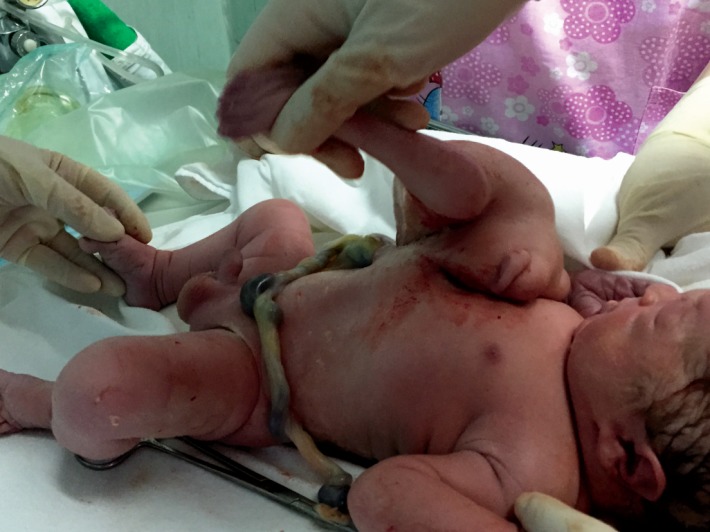
Asymmetric conjoined twins

**Fig. 10:**
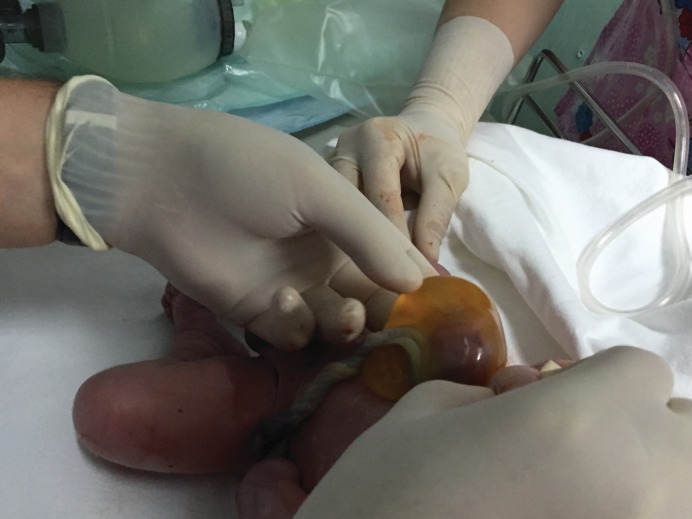
Asymmetric conjoined twins - Omphalocele

The viable fetus had a 40-millimeter omphalocele (**Fig. 10**). 

The newborn was transferred for further investigations and treatment to the pediatric surgery department of “Marie Curie” Children’s Hospital Bucharest. The result of the abdominal sonography of the autosite was in normal range. The heteropagus sonography revealed the existence of a unique kidney with hydronephrosis grade II, urinary bladder, diuresis and urinary incontinence. The echocardiography of the newborn showed a minimal membranous ventricular septal defect, atrial septal aneurysm with low-pressure coronary sinus dilation. Surgical separation was successful with an uneventful follow up postoperatively.

## Discussions

The incidence of multiple pregnancies is continuously growing, first as a consequence of assisted reproduction (3.9% of conjoined twins, 1 in 2 million live births) [**1**,**2**]. The pathogenesis of conjoined twins remains a mystery. Twin pregnancy with parasite fetus means that there is a multiply malformed fetus, its fetal parts or organs being attached to a viable fetus (autosite). The parasite is incompletely formed, small and completely dependent on the autosite [**7**]. One of the possible causes is the death in utero of a malformed fetus that remains attached and vascularized on the other twin (Spencer 2001) [**7**]. There are two categories of conjoined twins: symmetrical and asymmetrical. Heteropagus is the parasite attached to the abdomen of the viable fetus. 44 cases of epigastric heteropagus have been described in literature, a category that includes our clinical case [**3**]. The most preferable approach of this obstetrical pathology is the earlier beginning of the monitoring due to complications and the need of establishing an accurate diagnosis. Considering the fact that twin pregnancy with a parasite fetus is extremely rare, we considered the presentation of this case important and useful. 

From a pathogenetic standpoint, two possible theories were discussed: fusion and fission theory. The fusion hypothesis was the first to be proposed and it assumes an incomplete division of a single zygote, the formation of two cellular masses within the blastocyst at the end of the second week post conception. In consequence, a sole embrionary structure is formed, but with a single yolk sac. The DNA analysis always describes a homozygous karyotype [**4**,**5**]. 

The fission theory represents the incomplete division of the internal mass of the blastocyst followed by fusion on the 14-15 day post conception [**4**]. 

The ischemic atrophy is another proposed theory, in which the parasite twin is the result of an ischemic accident in the uterus, followed by the death and resorption of one of the fetuses, this event allowing the parasite twin to attach to the normally developed fetus [**4**,**5**]. 

The diagnosis methods are represented by conventional ultrasonography, Doppler, ¾ D and fetal MRI. 

In order to properly manage this type of medical case, it is of paramount importance to establish prenatal integrity of the vital organs and the anatomical and functional distribution of the shared organs between twins, which allows these cases a favorable postoperative outcome compared to the surgical approach of the symmetric conjoined twins. Unlike the symmetric conjoined twins, asymmetry associates a series of particular features: male gender is more frequent, the major vasa are not implicated, the digestive and skeletal systems are not described as possible connections between twins. The aforementioned clinical case had similar characteristics with the ones described in literature. The prognosis was excellent, the normally developed fetus having in general a favorable evolution after surgical separation [**6**]. However, there can be severe cardiac malformations on the viable fetus, which are frequently described in literature and were also found in our medical case. 

## Conclusions

Epigastric heteropagus is a rare condition, this presented case being the 45th described in literature, a fact that could contribute to the extension of the clinical overview, diagnosis and treatment of this particular pathology. These medical cases must be approached on a multidisciplinary basis, with an efficient collaboration between the obstetrician, neonatologist and pediatric surgeon in order to establish the proper time of birth, excluding the choice of a vaginal birth because it puts at risk both the viable fetus and the mother. Moreover, the prognosis highly depends on the understanding and the description of the anatomical particularities of the vascularity in these situations. 
